# A Manual of Personal Hygiene

**Published:** 1901-03

**Authors:** 


A MANUAL OF PERSONAL HYGIENE. Edited by Walter L. Pyle, A. M., M. D., Assistant Surgeon to the Wills Eye Hospital, Philadelphia; Fellow of the American Academy of Medicine; Former Editor of the *International Medical Magazine, etc.* Contributors: J. W. Courtney, M. D.; George Howard Fox, M. D.; E. Fletcher Ingals, M. D.; Walter L. Pyle, M. D.; B. A. Randall, M. D.; C. N. Stewart, M. D.; C. G. Stockton, M. D. Illustrated. Pp. 344; Price, $1.50. Philadelphia: W. B. Saunders & Co. 1900.

This book treats of a subject alike interesting to the layman and physician. It consists of an exposition of proper living upon a physiological basis. The language is simple and persuasive. How to eat, drink, breathe, bathe, sleep and take exercise so that the best results may come of it are subjects discussed in this book. Dr. Stockton, of Buffalo, wrote the article of the "Hygiene of the Digestive Apparatus." Dr. G. H. Fox, of New York, is the author of the portion of the work on the "Hygiene of the Skin and its Appendages." "Hygiene of the Vocal and Respiratory Apparatus" is by Ingalls, of Chicago. "Hygiene of the Ear" is by B. A. Randall, of Philadelphia. Dr. Pyle, besides editing the book, wrote the article on the "Hygiene of the Eye." The part devoted to the "Hygiene of the Brain and Nervous System" is from J. W. Courtney, of Boston. C. N. Stewart, of Cleveland, prepared the article on "Physical Exercise."

